# Hazards in Motion: Development of Mobile Geofences for Use in Logging Safety

**DOI:** 10.3390/s17040822

**Published:** 2017-04-10

**Authors:** Eloise G. Zimbelman, Robert F. Keefe, Eva K. Strand, Crystal A. Kolden, Ann M. Wempe

**Affiliations:** Department of Forest, Rangeland and Fire Sciences, University of Idaho, 875 Perimeter Drive, Moscow, ID 83844-1133, USA; robk@uidaho.edu (R.F.K.); evas@uidaho.edu (E.K.S.); ckolden@uidaho.edu (C.A.K.); awempe@uidaho.edu (A.M.W.)

**Keywords:** real-time positioning, GNSS-RF, GNSS, GPS-RF, GPS, geofence, virtual fence, situational awareness

## Abstract

Logging is one of the most hazardous occupations in the United States. Real-time positioning that uses global navigation satellite system (GNSS) technology paired with radio frequency transmission (GNSS-RF) has the potential to reduce fatal and non-fatal accidents on logging operations through the use of geofences that define safe work areas. Until recently, most geofences have been static boundaries. The aim of this study was to evaluate factors affecting mobile geofence accuracy in order to determine whether virtual safety zones around moving ground workers or equipment are a viable option for improving situational awareness on active timber sales. We evaluated the effects of walking pace, transmission interval, geofence radius, and intersection angle on geofence alert delay using a replicated field experiment. Simulation was then used to validate field results and calculate the proportion of GNSS error bearings resulting in early alerts. The interaction of geofence radius and intersection angle affected safety geofence alert delay in the field experiment. The most inaccurate alerts were negative, representing early warning. The magnitude of this effect was largest at the greatest intersection angles. Simulation analysis supported these field results and also showed that larger GNSS error corresponded to greater variability in alert delay. Increasing intersection angle resulted in a larger proportion of directional GNSS error that triggered incorrect, early warnings. Because the accuracy of geofence alerts varied greatly depending on GNSS error and angle of approach, geofencing for occupational safety is most appropriate for general situational awareness unless real-time correction methods to improve accuracy or higher quality GNSS-RF transponders are used.

## 1. Introduction

Logging involves frequent interactions among ground workers, heavy equipment, and dynamic terrain- and weather-related hazards, leading to many high-risk safety situations. As a result, logging is consistently ranked as one of the most hazardous occupations in the United States [1]. Fosbroke et al. analyzed national fatality data from the National Institute for Occupational Safety and Health (NIOSH) National Traumatic Occupational Fatalities (NTOF) surveillance system for the years 1990 and 1991 and found logging to have the highest lifetime risk of fatal injury [2]. Logging also has high rates of fatal and non-fatal injuries internationally [3,4]. The increasing mechanization of logging has been shown to reduce accident frequency, primarily by protecting workers in enclosed cabs from falling trees [5,6]. However, fatal injuries remain common, despite mechanization and stricter safety standards imposed by the U.S. Occupational Safety and Health Administration (OSHA) [7,8]. According to the U.S. Bureau of Labor Statistics, logging had the highest fatal work injury rate of civilian occupations in 2015 [9]. Direct contacts with trees and logs are responsible for a large portion of fatal injuries to loggers [1]. In particular, cable logging leads to many accidents resulting from manual falling with chainsaws, rolling logs, and close proximity of ground crews and heavy equipment. Low visibility between yarder operators and the ground crew contributes to many cable logging accidents [10].

Global navigation satellite system (GNSS) technology is used widely in positioning, navigation and timing (PNT). Utilizing real-time GNSS positioning information has the potential to mitigate hazards by enhancing communication and situational awareness on active timber sales. However, canopy cover in forested environments reduces GNSS accuracy, which has hindered widespread adoption of real-time positioning in forestry [11]. Sources of error in GNSS measurements include the number and arrangement of GNSS satellites as well as signal obstruction by forest canopy, topography, and buildings [12]. Of the three commonly recognized classes of GNSS receivers (survey-, mapping-, and recreation-grade), recreation-grade receivers are the least expensive, but also tend to provide the least accurate GNSS positional information [13]. We chose to work with a recreation-grade receiver because of the low cost and potential for widespread adoption. Previous studies have reported on the horizontal positional accuracy of stationary recreation-grade GNSS units. Using six recreation-grade GNSS units, Wing determined that the average error of the most accurate receiver was 2 m or less in open conditions, 3 m or less in young forest, and 9 m or less in closed canopy [14]. In a similar study, Wing calculated average errors of 2.5 m, 5.5 m, and 3.8 m in open, young, and mature stands, respectively [15]. Andersen et al. reported root mean squared error (RMSE) values of 3–7 m in a range of stand conditions in Alaska [16]. Bettinger and Fei calculated mean RMSE of 11.9 m and 6.6 m in young and mature loblolly pine stands, respectively, and 7.9 m in mature oak-hickory forest [13].

The expansion of GNSS services has led to the development of geofencing, which consists of delineating a geographic zone with a virtual perimeter [17]. This boundary can be a fixed radius around a designated point of interest or a polygon whose corners are defined by the user [18]. As tracked mobile objects move across the geofence, alarms may be signaled [18]. This system has great potential for monitoring the movement of people, equipment, vehicles and other mobile assets [19]. Currently, geofencing is used in fields such as transportation and logistics [19], fleet management [20], and mobile marketing and social networking [21]. Geofencing services have also been proposed for use in mobile tourism, through which visitors may receive personalized notifications based on their location within defined areas [22]. Song and Eldin proposed using geofencing to monitor heavy equipment and construction events in real-time on building construction and worksite operations [23]. Pestana et al. found that geofences can support airport operations by improving safety and efficiency [24] and Wawrzyniak and Hyla evaluated the use of geofencing technology to assist navigation of inland waterways [25]. Finally, in the defense and security sector, some governments have analyzed the potential for using geofences as tools to prevent terrorist attacks involving hazardous material transport [19].

In natural resources, geofence alerts have been suggested as a way to monitor fishing activity near exclusive economic zones (EEZ) [26] and as an indicator of marine protection areas [27]. Licht et al. described the use of geofences in real-time animal tracking [28] and Sheppard et al. proposed the use of geofencing to reduce bird mortalities at wind farms [29]. Wall et al. developed software for a real-time wildlife monitoring system that utilizes multiple movement algorithms [30]. In agriculture, Butler et al. developed a dynamic virtual fence algorithm as a fenceless method for herding cows [31] and Anderson et al. used a patented method called Directional Virtual Fencing to contain cattle within a moving virtual paddock [32]. Geofence techniques are also being considered to minimize potential collisions among Unmanned Aircraft Systems used in precision agriculture [33]. Other industries have investigated the use of real-time positioning and geofences for safety applications. Carbonari et al. developed a safety system for construction using ultra-wideband tracking and virtual fencing to warn workers of hazardous areas [34]. Information derived from GNSS units has been considered as a tool for improving operational monitoring and time studies in forest operations [35,36]. Combined global navigation satellite system-radio frequency transmission (GNSS-RF) systems integrate GNSS with radio transmission of location coordinates to facilitate position sharing among individuals and equipment working in the woods [37,38]. The use of multi transmitter GNSS-RF systems has been proposed for logging safety as well as for other applications in forest operations [37,38,39].

There are many situations on active logging operations, in wildland firefighting, transportation, recreation, and bioenergy fields in which it may be appropriate to deploy geofences around people, vehicles, equipment, animals, or other objects in motion, rather than around stationary objects or areas. For many of these safety applications in natural resources, the situational awareness of workers or recreationists could be improved if geofences were used in a mobile rather than stationary context. This is especially true in logging and wildland firefighting, two occupations that involve numerous interactions among ground workers, heavy equipment and dynamic terrain- and weather-related hazards. For example, mobile geofences could define safe working areas around manual fallers, log loaders and processors on ground-based logging operations, as well as around skyline carriages when using cable systems. When other ground workers or equipment operators wearing GNSS- or radio frequency identification (RFID)-enabled personal location devices (PLDs) pass into hazard zones delineated by geofences, alarms could be triggered so that equipment operators are aware of their presence. Mobile geofences might also be used to define early warning perimeters around log trucks to indicate their location driving on active haul roads or around vehicles carrying hazardous materials. Few studies have documented the accuracy of geofence alerts concerning their use in logging applications. Grayson et al. performed a replicated field experiment to determine how speed, intersection angle, and distance between the tracked object and GNSS receiver affect fixed location geofence accuracy using speeds corresponding to log trucks and skidders [38].

Until recently, geofences have primarily been used as static boundaries. A few exceptions include Guo et al., who developed a model for dynamic geofences centered on moving vehicles for use in accident prevention [40], as well as the algorithms described in Butler et al. and Anderson et al. [31,32]. There also is limited data on the effects of speed and transmission interval on mobile GNSS accuracy in forestry. Veal et al. measured the accuracy of tracking forest machines using GPS and found that equipment speed did not affect position accuracy [41]. However, the difference among equipment speeds evaluated was relatively small [41]. Grayson et al. found larger error associated with geofence crossing delays at slower speeds [38]. Piedallu and Gégout found that recording interval had a small effect on GPS accuracy and observed improved accuracy when moving from a 1-s to 5-s recording interval [42]. They observed smaller improvements in accuracy moving from 5-s to 10-s to 15-s recording intervals and this effect was mainly noticeable in closed canopy rather than in the open [42]. However, other studies have found either increases in location time [43], larger positional errors [44], or lower fix success rates [45] associated with longer intervals between recordings.

**Figure 1:**
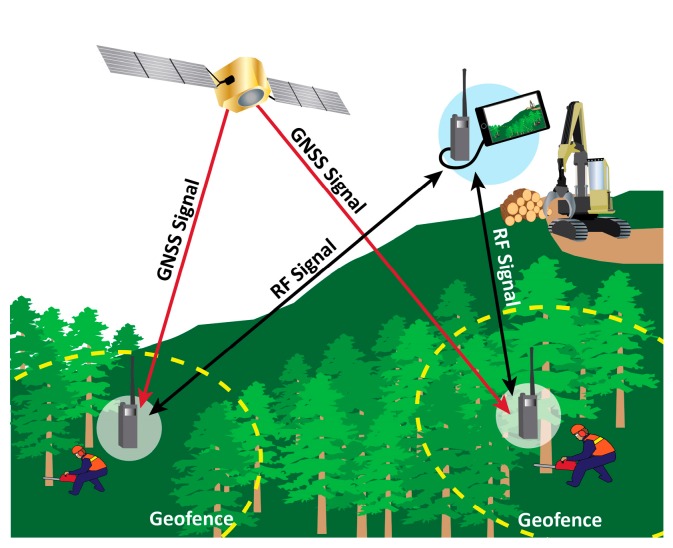
Illustration of the potential use of global navigation satellite system (GNSS) technology paired with radio frequency transmission (GNSS-RF) on a timber sale. GNSS-RF personal location devices (PLDs) receive positional information from GNSS satellites and send that information to nearby units using radio frequency transmission. In this figure, geofences with radii of approximately two tree lengths surround the manual fallers, delineating virtual perimeters associated with occupational hazards. Audible or sensory (e.g., vibration) alerts are triggered when other PLDs cross into the hazard areas, which move with individual workers.

In this paper, we expand on the concept of mobile geofences based on GNSS-RF technology for use in forestry as moving, circular safety zones around people and heavy equipment on active logging operations, a concept illustrated in Figure 1. We considered both traditional proximity alerts, as well as the overlap among multiple circular geofences of varying radii. To do this, a replicated experiment on the University of Idaho Experimental Forest was conducted to test the null hypothesis that walking pace, transmission interval, geofence size (radius), and the angle at which a geofence intersects a stationary point do not affect geofence alert delay. Our focus was primarily on the development of mobile safety geofences for manual fallers on logging operations due to the high number of fatal accidents that continue to occur during hand falling of timber. In order to provide a general method for analysis of mobile GNSS points in motion and associated geofences, simulation was used to describe the properties of circular geofences of varying sizes as they pass near one another or overlap, and the associated safety warning signals. We assumed GNSS error arises from a bivariate normal probability density. Our objective in doing so was to determine whether geofences and geofence alerts associated with recreation-grade GNSS-RF systems are suitable for occupational safety uses in remote, natural resource environments.

## 2. Materials and Methods

Two Garmin Alpha 100 handheld GNSS-RF units (Garmin, Olathe, Kansas, USA) were used with seven T5 transponders serving as PLDs to collect position measurements. T5 PLDs receive GNSS coordinates and relay them to handheld devices via radio frequency at user-defined intervals ranging from 2.5 to 120 s. The Garmin Alpha 100 units plot and record the positions of the PLDs, as well as their own positions at these same time intervals.

**Figure 2:**
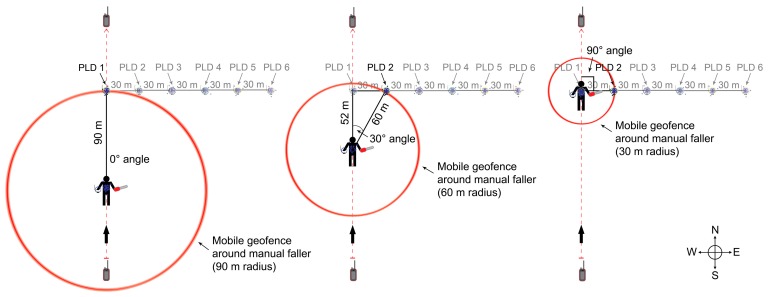
Illustration showing field experiment setup. The manual faller carried a T5 transponder (PLD) and a chainsaw along a 300-m route. Alpha 100 units recorded the GNSS data and were located at the start and end of the route. The six stationary PLDs located perpendicular to the route are shown. This figure shows the manual faller surrounded by mobile geofences of three radii, illustrating three possible intersection angles.

The experiment was carried out in Stand 358 of the East Hatter Creek unit of the University of Idaho Experimental Forest. Stand 358 has been managed using the seed tree regeneration method and was treated (harvested) in the year 2000. There were approximately 15–20 seed trees per hectare at 50-cm or greater diameter at breast height (DBH) in the residual overstory and 800 trees per hectare at 10-cm DBH in the regenerating cohort. The canopy was open and the terrain was gently rolling. Within the stand, a manual faller carrying a saw walked repeatedly along a fixed 300-m route with a T5 PLD recording the path, as shown in Figure 2.

The route was oriented north-south in order to minimize variation in the longitudinal coordinates associated with unit resolution. Six T5 PLDs were placed 1 m above the ground on wooden stakes perpendicular to the designated route. PLD 1 was located in the middle of the route 150 m from the start point. Moving due east from PLD 1, PLDs 2–6 were placed in 30-m increments. Two Alpha 100 handheld units mounted on wooden stakes 1 m above the ground recorded the PLD data during the experiment. One Alpha 100 was located at the start point and the second was at the end point. The experiment was an incomplete randomized block design. The manual faller walked along the route at three different paces as dictated by a digital metronome (30 bpm, 45 bpm, and 60 bpm), in combination with three different PLD transmission intervals (2.5 s, 5 s, and 10 s). The experiment was conducted over the course of three days, with each day representing one block. Each of the nine pace and transmission interval combinations appeared once per block and treatments were assigned at random. The manual faller was surrounded by seven different geofence radii (30, 45, 60, 75, 90, 105, and 120 m). These seven geofence radii combined with the six PLD locations described above resulted in 23 unique combinations of geofence radii and intersection angles, after excluding factor-level combinations that were not realized (Table 1). Intersection angle is the difference in azimuth formed between the vector heading of the walking path and the vector connecting the moving faller and stationary PLD at the time of intersection. Figure 2 shows three radius-angle combinations and illustrates how intersection angle is defined.

Beginning at the start point, the route was marked by brightly-colored pin flags every 10 m using a compass and 91-m fiberglass tape. Because the Garmin system does not support mobile geofencing, all of the locations at which the mobile geofence (at all seven radii) would first theoretically intersect each stationary PLD point were calculated. Prior to data collection, a fiberglass tape was used to flag these intersection points along the route. The locations of all flagged points along the route and of the six stationary PLDs were recorded at the beginning of the experiment using a survey-grade Topcon GR-3 GNSS base station and rover (Topcon Positioning Systems, Tokyo, Japan). This system has a specified accuracy of 10 mm. As the manual faller walked the route, the time at which he or she passed each flag was recorded using a custom script running in the R statistical programming environment [46] on a Windows tablet.

Intersection times recorded in the field represented the times at which the mobile geofence should have first intersected the stationary PLDs. The predicted times of intersection were calculated based on the ellipsoid distance between the GNSS coordinates recorded by the manual faller’s PLD and the stationary PLDs using the *distGeo* function in the R *geosphere* package [47]. In order to quantify the magnitude of signal delay, intersection times observed in the field were subtracted from the predicted intersection times using the following formula:

(1) $$D_{i}=P_{i}-O_{i},$$

where *D_i_* is the delay for the *i*th intersection, *P_i_* is the predicted intersection time for the *i*th intersection calculated using the recorded GNSS coordinates, and *O_i_* is the field-recorded National Institute of Standards and Technology (NIST) time when the faller crossed the *i*th intersection point.

Using this method, positive time delays indicate geofence crossing alerts that would have occurred after the faller had actually crossed the geofence. Likewise, negative delays represent alerts that would have been triggered prior to the faller crossing the geofence. A linear mixed-effects model was used to account for correlation within each block and within each pass along the route because the assumption of within-block independence of errors was not satisfied for conventional analysis of variance (ANOVA). The mixed-effects model was fit using the *lme* function in the R *nlme* package [48]. The model was fit using all factor-level combinations as fixed effects and the block and pass along the route as random effects. The pass along the route was nested within each block. The error correlation was incorporated using an autoregressive structure. ANOVA was used to determine the significance of the fixed effect terms in the model. Normality was an issue with the initial model, so a square root transformation was applied to the intersection delay such that negative delays remained negative and positive delays remained positive.

A simulation script was also written in R to evaluate the geofence alert delay as a mobile geofence intersected a stationary geofence. Eight mobile geofence radii (50, 60, 70, 80, 90, 100, 110, and 120 m) were combined with 91 stationary PLD locations (0–180 m, in 2-m increments) perpendicular to the midpoint of a 500-m route. Each stationary PLD was surrounded by a 50-m radius geofence. The center point of the mobile geofence was advanced in 2-m increments along the route, with 1000 cycles per treatment. Both the fixed PLD locations and the moving center point of the mobile geofence were assumed to have bivariate Gaussian probability density, such that their intersection was a mixture of the two distributions. This error was applied in R using five standard deviations (1, 2, 3, 4, and 5 m) representing varying levels of GNSS accuracy. To characterize the simulation results, the *nls* function in R [46] was used to fit a non-linear exponential model of the following form:

(2) $$D_{ijkl}=\beta_{0}+\beta_{1}*e^{\left(\beta_{2}*a_{j}\right)}+\beta_{3}*s_{k}+\beta_{4}*r_{l}+\varepsilon_{i},$$

where *D_ijkl_* is *i*th time delay, *a_j_* is the *j*th intersection angle, *s_k_* is the *k*th standard deviation, and *r_l_* is the *l*th geofence radius.

To better understand the results from both the field experiment and simulation, the proportion of error bearings resulting in early alerts was calculated for the intersection of a mobile geofence with a stationary geofence. We assumed that a GNSS point is equally likely to be moved in 360 directions (bearings) from its true location due to error. Thus, a certain proportion of those potential errors might move the point (and thus the geofence surrounding it) in a way that would cause an early intersection alert. This proportion depends on geofence radius, approach angle, true distance between the two GNSS points, and error magnitude. For our calculations, both geofences had 50-m radii. The mobile and stationary geofences were oriented such that the angle at the true intersection point would be 0°, 22.5°, 45°, 67.5°, and 90°. Within each of these five approach orientations (intersection angles), we placed the mobile geofence at four starting locations (1, 4, 7, and 10 m from the true intersection point) and moved each three distances (1, 3, and 5 m) from this initial location along vectors with bearing angles from 0° to 359°. For each factor-level combination, the proportion of these 360 error angles that resulted in an early alert was calculated.

## 3. Results

### 3.1. Field Results

Incorporating an autoregressive correlation structure into the mixed-effects model of the field results resulted in an improved model with a lower Akaike Information Criterion (AIC) and lower log likelihood (*p* < 0.0001) (Table 2). ANOVA on this model showed that the interaction between radius-angle combination and pace (*p* < 0.0001) as well as the radius-angle combination (*p* < 0.0001) affected intersection delay. However, the assumption of normality was not met so the model was refit using a square root transformation of the delay. In the resulting model, ANOVA indicated that only the radius-angle combination affected intersection alert delay (*p* < 0.0001) (Table 3).

**Figure 3:**
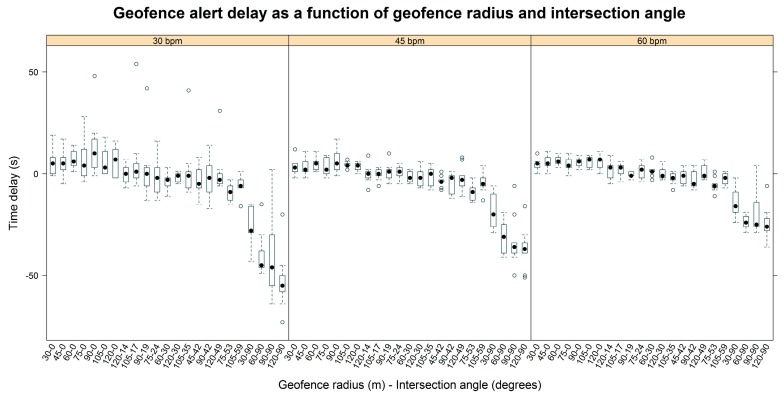
Box-and-whisker plot of field results showing geofence intersection alert delay as a function of the geofence radius-intersection angle combinations. The panels are grouped by pace (30, 45, and 60 bpm).

Alert delay for the entire field experiment ranged from −73 s to 54 s. The most inaccurate alerts tended to be negative and were observed at the slower paces and largest angles. Figure 3 illustrates the geofence alert delay as a function of the radius-angle combinations grouped by the three levels of walking pace (30, 45, and 60 bpm). It shows a more negative alert delay, meaning an earlier alert, as the angle of intersection increases. There also appears to be greater variability in the intersection alert delay at slower paces. Finally, the alert delay is less negative at faster paces with the most negative delays occurring at the slowest pace at the largest angles. These more negative delays correspond to earlier hazard alerts.

**Figure 4:**
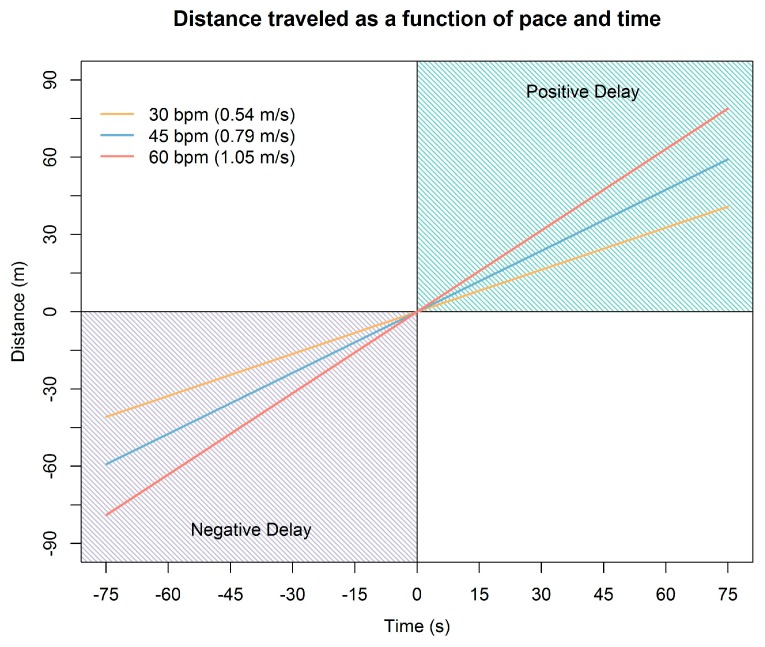
Distance traveled over time as a function of walking pace. The slope of each line corresponds to the mean observed speed for each level of pace. Positive distances indicate how far the faller has walked into or past the geofence boundary when the alert is generated (i.e., a positive, late warning). Negative distances indicate how far ahead of the intersection point the faller is when the alert is generated (i.e., a negative, early warning).

To relate the magnitude of alert delay to distance, Figure 4 shows the distance traveled over time as a function of the mean observed speed for each level of walking pace used in the field experiment. For the most positive delay observed (54 s), the faller would have walked 29.4, 42.6, or 56.8 m past the geofence at the 30-, 45-, and 60-bpm paces, respectively. For the most negative delay observed (−73 s), an alert would have been received when the faller was 39.8, 57.6, or 76.8 m from reaching the geofence boundary at the 30-, 45-, and 60-bpm paces, respectively.

### 3.2. Simulation Results

**Figure 5:**
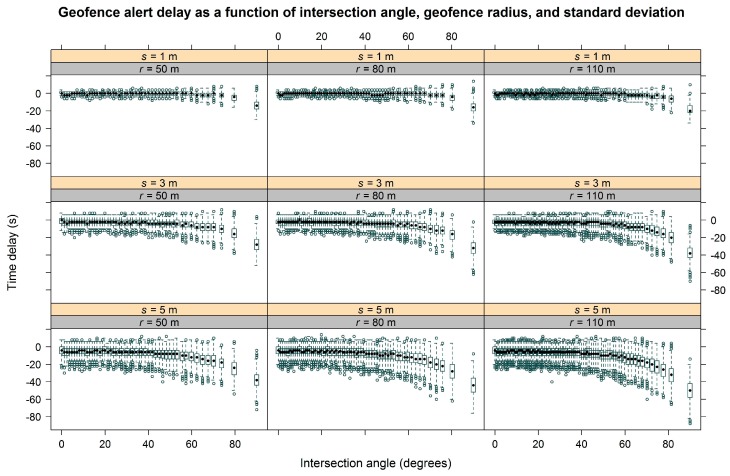
Box-and-whisker plot of simulation results showing geofence alert delay as a function of intersection angle grouped by radius and GNSS standard deviation. To improve clarity, the figure is a subset of factor-level combinations, representing three geofence radii (*r* = 50, 80, and 110 m) and three standard deviations (*s* = 1, 3, and 5 m). Upper panel numbers are GNSS standard deviation and lower panels represent geofence radii.

Simulation analysis supported field results, illustrating the effect of intersection angle on geofence alert delay. Figure 5 shows the geofence alert delay as a function of intersection angle grouped by radius and GNSS standard deviation. There is more error in the alert delay associated with higher standard deviations and there is a general trend toward more negative delays (earlier alerts) as standard deviation increases. This trend is most noticeable at the larger angles. Also, more negative delays (earlier alerts) occur as intersection angle increases. This pattern is clearest at the larger standard deviations. Finally, in terms of geofence radius, slightly more negative delays were associated with larger radii at the largest intersection angles.

Table 4 shows the results from the exponential model fitted to the simulation data. All model coefficients had *p*-values less than 2 × 10^−16^.

**Figure 6:**
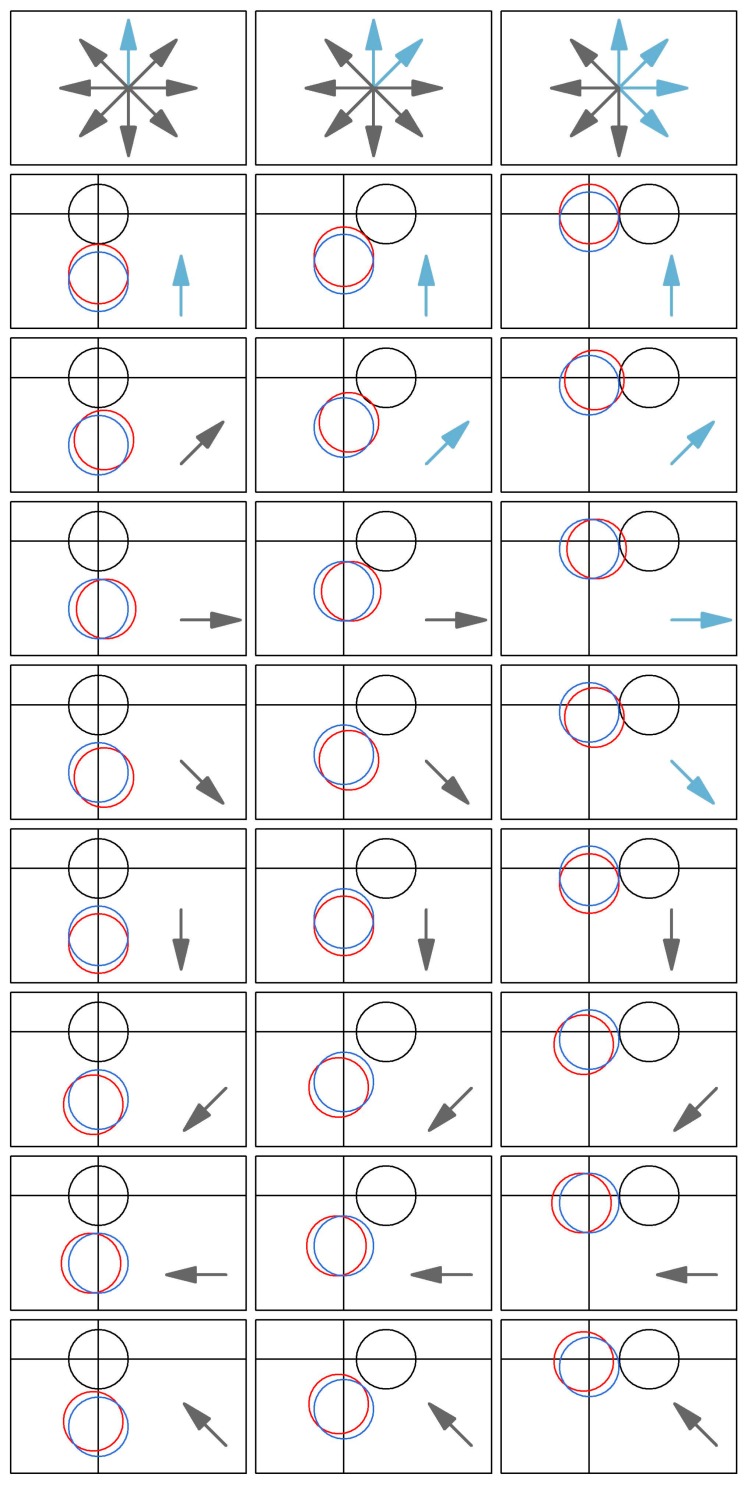
Visualization of GNSS error bearings resulting in early alerts. In each cell, the black circle is a stationary geofence and the blue circle represents a mobile geofence located 20 m from its initial intersection point with the stationary geofence. In the first column, the geofence intersection angle will be 0°. In the second and third columns, the intersections will occur at 45° and 90°, respectively. The red circle in each cell illustrates the location of the mobile geofence if it were moved 20 m from its true location, with each row representing one of eight possible directions in which that 20-m error might occur. Arrows in each cell indicate the directionality of error, with light blue arrows indicating movements that result in early intersection alerts. Black arrows indicate movements that do not result in early intersection alerts. The top row depicts all eight error directions in each column below and summarizes which of those directions result in early alerts.

Figure 6 illustrates how the bearing angle at which error occurs triggers early alerts depending on the intersection angle of stationary and mobile geofences. It is evident in the figure that there is an interaction among circle geometry and the directionality of GNSS error that affects alert timing. There are more possible error bearing angles that result in early alerts as intersection angle increases from 0° to 90°.

**Figure 7:**
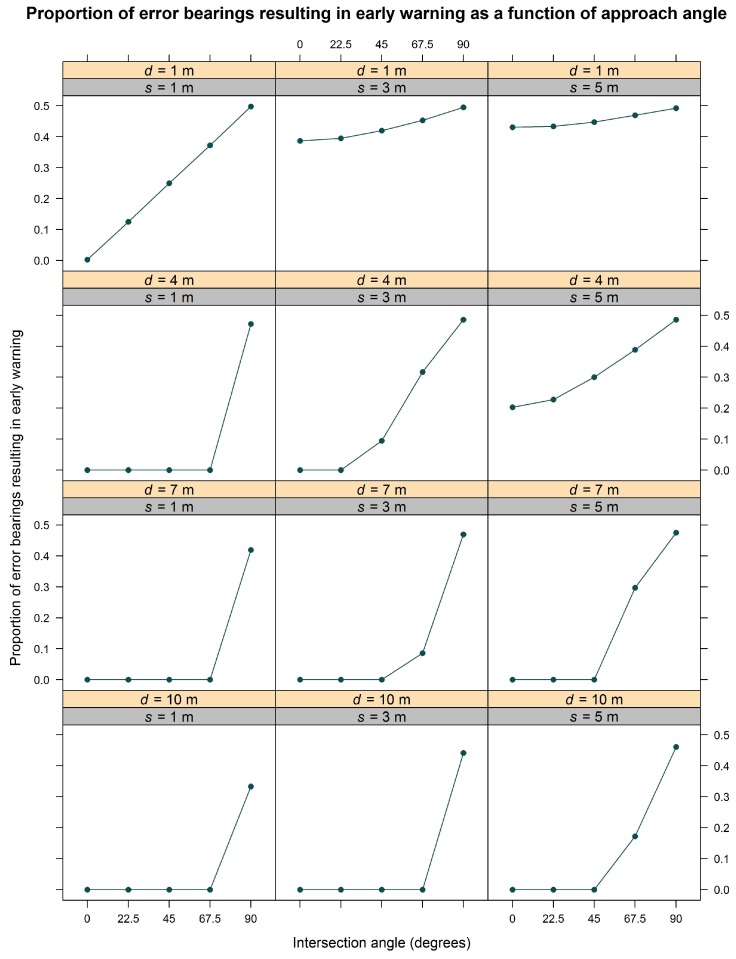
Proportion of error bearing angles that result in early alert plotted as a function of the intersection angle. Calculations were done for five intersection angles (0°, 22.5°, 45°, 67.5°, and 90°). The upper panel labels represent the four starting locations (*d* = 1, 4, 7, and 10 m from the true intersection point) and the lower panels are the three GNSS standard deviations (*s* = 1, 3, and 5 m).

To better demonstrate this effect, additional simulation results shown in Figure 7 illustrate the proportion of error bearing angles that result in early alert as a function of the intersection angle grouped by four starting locations (1, 4, 7, and 10 m from the true intersection point) and three GNSS standard deviations (1, 3, and 5 m). In all cases, the 90° angle has the highest proportion of directional error movements that result in early warning. The difference in this proportion is greatest when the starting location is further from the intersection point. The difference in proportions also varies indirectly with the magnitude of GNSS standard deviation. This is most noticeable at the closest starting locations.

## 4. Discussion

Results from the field experiment indicate that the realized geofence radius-intersection angle combinations had a significant effect on the alert delay. Similarly, simulation results illustrated the effect of intersection angle on alert delay. Because of the way in which intersection angles were calculated, a 0° angle means the mobile geofence approached the PLD or stationary geofence straight-on, while a 90° angle means the mobile geofence approached alongside the PLD or stationary geofence. In both the field experiment and the simulation, the delay was closest to 0 s at smaller angles. As the angle increased to 90°, delay decreased (became more negative), meaning that an earlier alert was signaled. This effect appears to result from a phenomenon that is evident in Figure 7, in which the proportion of directional GNSS error that triggers an early warning depends on angle of intersection approach. In other words, when a mobile geofence approaches a point of interest (PLD) straight-on (i.e., at a 0° angle), only a small proportion of all potential GNSS error results in an early warning. However, when a mobile geofence approaches alongside a PLD (i.e., at a 90° angle), a larger proportion of all potential GNSS error results in early warning. This result has important implications for use of mobile geofences in logging safety because approach angles are constantly changing on active timber sales. This means the accuracy of any alert will vary as worker or equipment positions move around one another at different angles. For instance, if a worker surrounded by a mobile geofence moves straight toward a piece of equipment, the intersection alert may occur with relatively high accuracy. However, if the same worker were to approach alongside the equipment, intersection alert generation may have lower accuracy.

Because of the geofence radii and PLD locations used in the field experiment, not all factor-level combinations of geofence radius and intersection angle were realized. However, simulation results indicate the effect intersection angle has on alert delay, independent of radius. On the other hand, the effect of radius is not as clear. It seems that larger geofence radii trigger slightly earlier alerts at the largest angles but future research is necessary in order to understand this relationship more clearly.

In the field experiment, the delay was more negative at the slowest pace (30 bpm) and became closer to 0 s at the fastest pace (60 bpm). This was primarily noticeable at the largest intersection angles. While this effect was not statistically significant in the mixed-effects model, a moderate influence of pace seems evident graphically in Figure 3. A clearer effect of pace on geofence alert timing was observed previously in Grayson et al. [38]. Transmission interval also did not affect delay in the mixed-effects model used to analyze field results, and there were no noticeable trends in delay at the three transmission intervals evaluated. This result is counterintuitive and may be a consequence of the range of intervals considered.

To continue to advance the use of mobile geofences for delineation of safe work zones in logging and other hazardous occupations with many moving parts, correction methods that account for the effect of intersection angle, and possibly other factors, may be needed. In our study, the absolute point at which GNSS-RF transponders crossed geofence boundaries was assumed to be the appropriate time for a hazard alert to be triggered. However, in practice, alerts would be triggered at earlier warning thresholds in order to provide information about approaching hazards with sufficient time for equipment operators or ground workers to slow down or change course. In further developing the use of mobile geofences to define hazard boundaries and associated safe work areas around workers and equipment, the effect of intersection angle identified in this study will need to be coupled with use of early warning thresholds based on the speed and distance relationships shown in Figure 4.

Although the focus of this study was on use of mobile geofences as tree falling hazard zones around manual fallers, there are many other potential safety applications on active logging operations for which mobile geofences could be deployed. For example, mobile geofences could be used to indicate the relative locations of ground workers working at the log landing adjacent to loading and processing equipment, or to indicate the relative proximity of rigging crew workers to the skyline carriage on cable logging operations. As with self-driving vehicle technology used in other fields, an eventual application of mobile geofences to indicate and reduce work-related hazards may be the use of technology not only to provide warnings to ground workers or equipment operators, but to slow or stop equipment function when occupational hazards are imminent.

## 5. Conclusions

Logging continues to be one of the most dangerous occupations in the United States. Real-time positioning has the potential to improve communication and situational awareness on active timber sales. Geofences are an important component of real-time positioning systems. While mobile geofences may be useful in alerting workers when they get too close to jobsite hazards, the accuracy of these alerts may vary depending on angle of approach and GNSS accuracy. Because accuracy and intersection angle change frequently on active logging operations, relying on mobile geofences without application of correction methods is not advised for fine resolution delineation of safe work areas. However, uncorrected mobile geofences may still be appropriate for increasing general situational awareness among workers at coarser spatial scales. A better understanding of the effects of these factors could inform the development of correction methods to improve alert accuracy and recommendations for use of real-time positioning technology in occupational safety.

## Figures and Tables

**Table 1:** Table showing the seven different mobile geofence radii and their intersections with the six different PLDs. The resulting intersection angles are shown.

PLD	Radius (m)	Angle (Degrees)
1	30	0
2	30	90
1	45	0
2	45	42
1	60	0
2	60	30
3	60	90
1	75	0
2	75	24
3	75	53
1	90	0
2	90	19
3	90	42
4	90	90
1	105	0
2	105	17
3	105	35
4	105	59
1	120	0
2	120	14
3	120	30
4	120	49
5	120	90

**Table 2:** Analysis of variance (ANOVA) results comparing models with and without autoregressive error structure. Model 1 was fit without correlated errors while Model 2 was fit with autoregressive error structure.

Model	DF ^1^	AIC ^2^	BIC ^3^	Log Lik ^4^	Test	L Ratio ^5^	*p*-Value ^6^
1	210	4394.911	5325.49	−1987.456	-	-	-
2	211	4162.837	5097.848	−1870.418	1 vs. 2	234.074	<0.0001

^1^ Model degrees of freedom; ^2^ Akaike Information Criterion; ^3^ Bayesian Information Criterion; ^4^ Restricted log likelihood; ^5^ Likelihood ratio; ^6^ *p*-value associated with likelihood ratio statistic.

**Table 3:** ANOVA results from the full model showing all main effects and interactions. The response was the square root of the alert delay and the model was fit using autoregressive error structure.

Model Term	Num DF ^1^	Den DF ^2^	*F*-Statistic ^3^	*p*-Value ^4^
(Intercept)	1	396	8.43367	0.0039
Rad Ang	22	396	76.00023	<0.0001
Pace	2	16	1.24997	0.313
TI	2	16	1.16111	0.3382
RadAng: Pace	44	396	0.937	0.5896
RadAng: TI	44	396	0.83993	0.7571
Pace: TI	4	16	0.60202	0.6667
RadAng:Pace: TI	88	396	1.24072	0.0874

^1^ Numerator degrees of freedom; ^2^ Denominator degrees of freedom; ^3^ *F*-statistic for Wald tests for model terms; ^4^ *p*-value associated with Wald tests for model terms.

**Table 4:** Summary of the exponential model (Equation (2)) fitted to the simulation results showing the estimate of each model coefficient, standard errors and *p*-values.

Parameter	Estimate ^1^	Std. Error ^2^	*t*-Statistic ^3^	*p*-Value ^4^
b0	3.86 × 10^0^	1.35 × 10^-2^	286.41	<2 × 10^−16^
b1	−5.79 × 10^-2^	3.64 × 10^-4^	−159.06	<2 × 10^−16^
b2	6.92 × 10^-2^	7.33 × 10^-5^	944.50	<2 × 10^−16^
b3	−2.00 × 10^0^	2.03 × 10^-3^	−985.52	<2 × 10^−16^
b4	−4.29 × 10^-3^	1.27 × 10^-4^	−33.76	<2 × 10^−16^

^1^ Estimated model coefficient; ^2^ Standard error of estimated model coefficient; ^3^ *t*-statistic for each model coefficient; ^4^ *p*-value associated with the *t*-statistic for model coefficients.
